# Application of density estimation algorithms in analyzing co-morbidities of migraine

**DOI:** 10.1007/s13721-013-0028-8

**Published:** 2013-02-12

**Authors:** Meng-Han Yang, Fu-Yi Yang, Yen-Jen Oyang

**Affiliations:** 1 Department of Computer Science and Information Engineering, National Kaohsiung University of Applied Sciences, No. 415 Chien Kung Rd., Kaohsiung, 80778 Taiwan, ROC; 2 The Department of Neurology, Taipei Tzu Chi General Hospital, No. 289, Jianguo Rd., Xindian District, New Taipei, 23142 Taiwan, ROC; 3 Department of Computer Science and Information Engineering, National Taiwan University, No. 1, Sec. 4, Roosevelt Rd., Taipei City, 10617 Taiwan, ROC

**Keywords:** Density estimation algorithm, Migraine, Drug treatment, Co-morbidity

## Abstract

In this study, we will propose a density estimation based data analysis procedure to investigate the co-morbid associations between migraine and the suspected diseases. The primary objective of this study has aimed to develop a novel analysis procedure that can discover insightful knowledge from large medical databases. The entire analysis procedure consists of two stages. During the first stage, a kernel density estimation algorithm named relaxed variable kernel density estimation (RVKDE) is invoked to identify the samples of interest. Then, in the second stage, a density estimation algorithm based on generalized Gaussian components and named G^2^DE is invoked to provide a summarized description of the distribution. The results obtained by applying the proposed two-staged procedure to analyze co-morbidities of migraine revealed that the proposed procedure could effectively identify a number of clusters of samples with distinctive characteristics. The results further revealed that the distinctive characteristics of the clusters extracted by the proposed procedure were in conformity with the observations reported in recently published articles. Accordingly, it is conceivable that the proposed analysis procedure can be exploited to provide valuable clues of pathogenesis and facilitate development of proper treatment strategies.

**Electronic supplementary material:**

The online version of this article (doi:10.1007/s13721-013-0028-8) contains supplementary material, which is available to authorized users.

## Introduction

In recent years, data analysis based on large medical and clinical databases has gained attention among biomedical researchers (Himes et al. 2009; Lai et al. 2010; Lugardon et al. 2007). One major merit of this type of studies is that these databases collect cases with good demographic diversity. In addition, researchers can expeditiously verify their hypotheses since they do not need to spend a significant amount of efforts to recruit cases. Nevertheless, most studies have been conducted with conventional bio-statistical approaches. Accordingly, scientists have turned to exploit advanced machine learning and/or data mining approaches to extract valuable clues hidden in large medical and clinical databases (Himes et al. 2009; Lancashire et al. 2005; Li et al. 2004; Niederkohr and Levin 2005). For example, the Bayesian network has been exploited to identify the co-morbidity between chronic obstructive pulmonary disease and asthma (Himes et al. 2009). Furthermore, the decision tree algorithm has been exploited to guide diagnostic interpretation and therapeutic options for temporal arteritis (Niederkohr and Levin 2005).

In our study, we have aimed to exploit density estimation algorithms in the analysis of large medical/clinical databases. Density estimation is a classical problem in statistics aimed at constructing an approximate probability density function based on the samples randomly and independently taken from an underlined distribution. In the proposed approach, we have exploited the relaxed variable kernel density estimation (RVKDE) algorithm (Oyang et al. 2005) and the generalized Gaussian component based density estimation (G^2^DE) algorithm (Hsieh et al. 2009) that our research team has developed in recent years. The RVKDE algorithm has been exploited to identify those case samples that share some distinctive features in comparison with the control samples. Then, the G^2^DE algorithm has been invoked to provide a summarized and highly interpretable description of the underlying distribution.

In our study, aiming to learn the actual effects of the proposed analysis procedure, we have applied the proposed procedure to analyze co-morbidities of migraine. Migraine is a prevalent neurological disorder whereby patients suffer from recurrent headache attacks, nausea, photophobia, and phonophobia. Recent demographical studies showed that migraine was more common to women than to men and its burden has been underestimated. Many illnesses, physical or psychiatric, have been reported to be co-morbid with migraine (Aamodt et al. 2007; Bigal et al. 2010; Buse et al. 2010; Hagen et al. 2002; Kurth et al. 2008; Le et al. 2011); these disorders occur at a greater coincidental rate among migraine patients than among the general population. Understanding the association of migraine with other health conditions can help the clinicians providing better care and investigate the pathogenesis of these disorders.

## Methods

### Density estimation algorithms

In this section, we will elaborate the main features of the RVKDE algorithm and the G^2^DE algorithm exploited in the proposed analysis procedure and the desired effects achieved. Basically, the RVKDE algorithm was designed to construct an approximate probability density function with high accuracy. On the other hand, the G^2^DE algorithm was designed to provide a summarized and highly interpretable description of the underlying distribution.

Let {*s*_1_, *s*_2_, …, *s*_*n*_} be a set of samples randomly and independently taken from the distribution governed by probability density function *f* in a *d*-dimensional vector space. Then, the RVKDE algorithm constructs an approximate probability density function $\hat{f}$ based on the following general form:

1 $$ \hat{f}({\mathbf{v}}) = \frac{1}{|n|}\sum\limits_{{s_{i} }} {\left( {\frac{1}{{\sqrt {2\pi } \cdot \sigma_{i} }}} \right)^{d} \exp \left( { - \frac{{||{\mathbf{v}} - s_{i} ||^{2} }}{{2\sigma_{i}^{2} }}} \right)} $$

where $\sigma_{i} = \beta \frac{{R(s_{i} )\sqrt \pi }}{{\root{d} \of {{(k + 1)\Upgamma (\tfrac{d}{2} + 1)}}}}$, *R*(**s**_*i*_) is the maximum distance between **s**_*i*_ and its *k* nearest training instances; Γ(·) is the gamma function (Artin 1964); *β* and *k* are parameters to be set either through cross validation or by the user.

The general form of the RVKDE algorithm indicates that, for each sample, a Gaussian function is placed at its corresponding coordinates in the vector space. Accordingly, the approximate function constructed by the RVKDE algorithm is composed of a large number of Gaussian functions and it is difficult for a user to gain an abstract image of the underlying distribution in a multiple-dimension vector space. Therefore, our research team has designed the G^2^DE algorithm to provide the complementary feature. The approximate function constructed by the G^2^DE algorithm is composed of a limited number of generalized Gaussian components as shown in the following:

2 $$ \hat{f}(v) = \frac{1}{{\sum {w_{i} } }}\sum\limits_{i = 1}^{k} {\frac{1}{{\left( {2\pi } \right)^{d/2} }}{\text{GGC}}\; (w_{i} ,\mu_{i} ,\Upsigma_{i} )} $$

where ${\text{GGC}}\; (w_{i} ,\mu_{i} ,\Upsigma_{i} )= w_{i} \frac{1}{{\left| {\Upsigma_{i} } \right|^{1/2} }}\exp \left( { - \frac{1}{2}(v - \mu_{i} )^{T} \sum\nolimits_{i}^{ - 1} {(v - \mu_{i} )} } \right)$, *d* is the dimension of the vector space, $w_{i} ,\mu_{i} ,\;{\text{and}}\;\Upsigma_{i}$ are the weight, center, and the covariance matrix of the *i*-th Gaussian component, respectively.

Since each Gaussian component in a G^2^DE based probability model corresponds to a cluster of samples, we can examine the centers and the covariance matrices of the Gaussian components to obtain an abstract image of the underlying distribution. Nevertheless, it must be noted that the number of parameters in a G^2^DE based probability model is equal to $\frac{k(d + 2)(d + 1)}{2}$. As a result, if we do not set *k* and *d* to small integers, then we need to examine a large number of parameter values and it may be difficult for us to interpret the physical meanings of the parameter values.

### The clinical database

The study reported in this article has been conducted based on the Research Database released by the National Health Insurance Program in Taiwan. The National Health Insurance (NHI) program in Taiwan was launched in 1995 and as in December 2010 covered about 23,074,000 insurants, which accounted for over 99 % of the entire population in Taiwan. In addition, almost all medical hospitals and clinics in Taiwan have joined the program. As in December 2010, there were 25,031 medical institutes enrolled in the program. Since 2000, the Bureau of the program began to release the National Health Insurance Research Database (NHIRD) to facilitate medical research. The updated version used in this study contains the ambulatory and hospitalization claims records of 1,000,000 randomly selected insurants over the period from 1996 to 2010 without significant difference in age, sex, and insurance cost relative to the whole population.

### Case patient definition and control selection

The cases in this study include those patients who were diagnosed with migraine in outpatient and/or inpatient records during 2004–2008. The ICD-9 CM codes (International Classification of Disease, 9th Revision, Clinical Modification; http://icd9cm.chrisendres.com/) used for screening include 346.0×, 346.1×, 346.8×, and 346.9×, which correspond to patients with migraine with or without aura. In our study, for each migraine case, five controls without any migraine record during 1996–2010 and with matched gender and age were randomly selected from the NHIRD. As a result, the cohort contained 19,356 migraine cases and 96,780 controls. For a case, the date of the first migraine diagnosis was defined to be the index date and the same index date was assigned to the matched controls.

### Medication exposure utilized as features

In our analysis, each cohort subject was associated with a feature vector that recorded the exposure of the subject to the commonly used medications for migraine treatment during the study period, including amitriptyline, flunarizine, propranolol, topiramate, and valproic acid. The exposure was measured by the number of days and the dosage in milligrams. The dosage was also calculated in defined daily dose (DDD) by World Health Organization (http://www.whocc.no/atc_ddd_index/) for validation. The exposure to each category of medications was counted separately. Accordingly, the feature vector is composed of ten elements. In our analysis, we further normalized the feature values corresponding to the same element in the feature vector by applying the standard min–max normalization.

The five categories of drugs for migraine treatment mentioned above all belong to preventive medicines. Aiming to validate drug medications of our study population, we also analyzed the prescription orders for ergotamine during the study period, which is a frequent relief treatment of migraine attacks.

### Diseases utilized as outcomes

Our study focused on those diseases that had been reported to be the co-morbidities of migraine (Aamodt et al. 2007; Bigal et al. 2010; Buse et al. 2010; Hagen et al. 2002; Le et al. 2011). These diseases can be classified into six categories as follows based on the ICD-9 CM codes:

1. Mental disorders: alcohol abuse (ICD-9 CM codes: 265.2, 291.xx, 303.xx, 305.0x, 357.5, 425.5, 535.3x, 571.0, 571.1, 571.3, 980.x, and V113); anxiety state (codes: 300.00, 300.02, and 300.09); bipolar disorder (codes: 296.0x, 296.1x, 296.4x, and 296.6x–296.9x); depression (codes: 296.2x, 296.3x, 296.5x, 300.4, 309.xx, and 311); drug abuse (codes: 292.xx, 304.xx, 305.2x–305.9x, and V6542); psychoses (codes: 293.8x, 295.xx, 297.x, and 298.x)
2. Otolaryngology: allergic rhinitis (ICD-9 CM codes: 477.x); chronic pulmonary disease (codes: 490–496, 500–505, and 506.4); Meniere’s disease (codes: 386.0x)
3. Musculoskeletal illnesses: low back pain (ICD-9 CM codes: 724.xx); neck pain (code: 723.1); neck sprain (code: 847.0); pain syndrome (codes: 719.4x and 729.1); rheumatoid arthritis (codes: 446.x, 701.0, 710.2, 710.3, 710.8, 710.9, 711.2x, 714.3x, 714.4, 714.89, 714.9, 719.3x, 720.xx, 728.5, 728.89, and 729.30); spinal disk herniation (codes: 722.0–722.2, and 722.7x);
4. Metabolism and endocrinology: diabetes mellitus (ICD-9 CM codes: 250.0x–250.3x, and 250.7x); fluid electrolyte disorder (codes: 253.6 and 276.x); hyperlipidemia (codes: 272.x); hypothyroidism (codes: 240.9, 243, 244.x, 246.1, and 246.8); obesity (codes: 278.0x);
5. Cardiovascular and neurological diseases: cardiac arrhythmias (ICD-9 CM codes: 426.0, 426.1x, 426.7, 426.9, 427.0–427.4x, 427.6x, 427.8x, 427.9, 785.0, 996.01, 996.04, V45.0x, and V53.3x); cerebrovascular disease (codes: 430–438.xx); coronary artery disease (codes: 410.xx–414.xx); heart failure (codes: 428.x); hypertension (codes: 401.x); peripheral vascular disease (codes: 441.9, 443.9, 785.4, and V434); epilepsy (codes: 345.xx)
6. Gastroenterology and hepatology: kidney stone (ICD-9 CM codes: 592.0); liver disease (codes: 571.2, 571.4x–571.6); peptic ulcer disease (codes: 531.xx–534.xx); renal disease (codes: 582.xx, 583.0–583.2, 583.4, 583.6, 583.7, 585, 586, and 588.x).

For each subject, outpatient and/or inpatient diagnoses of these disorders during the study period would be analyzed. Demographics and clinical variables were compared between migraine cases and controls using the Chi-square test or student’s *t* test when appropriate. We have employed the odds ratio (OR) with 95 % confidence interval to quantify the risk of a co-morbidity of migraine in different groups of patients. All tests were two-tailed, and *p* values of <0.05 were considered significant.

### The analysis procedure

The analysis procedure consists of two stages. During the first stage, the RVKDE algorithm was invoked to construct one approximate probability density function for the cases, denoted by $\hat{f}$, and another probability density function for the controls, denoted by $\hat{f}^{\prime }$. Then, all the cases were examined one by one. Let ${\mathbf{s}}_{i}$ denote the feature vector corresponding to the *i*-th case in the dataset. If $\hat{f}({\mathbf{s}}_{i} )/\hat{f}^{\prime } ({\mathbf{s}}_{i} )$ is greater than a threshold, then the case was labeled as sample of interest. As mentioned earlier, this screening process aimed to identify those cases that shared some distinctive features in comparison with the controls.

During the second stage, the G^2^DE algorithm was invoked to cluster the cases of interest and provided summarized descriptions of the clusters. However, as mentioned earlier, the number of features, which correspond to the dimension of the vector space and thus the dimension of the covariance matrix output by the G^2^DE algorithm, should be limited to a small integer for us to easily obtain an abstract image of the underlying distribution. Accordingly, we incorporated a feature selection process before invoking the G^2^DE algorithm. The feature selection process proceeded as follows. First, the correlation matrix of the original ten features is derived based on the cases of interest identified in the first stage of analysis. Then, those eigenvectors with the corresponding eigenvalue larger than 1 are selected to form the factor space. Finally, the factor space is rotated orthogonally and the component features of the rotated factors with a loading larger than 0.4 are selected to form a subspace into which the original dataset is projected.

## Results

Table 1 shows the demographics of the entire dataset, which includes 19,356 migraine cases and 96,780 controls. As expected, the distributions of ages and genders are identical among migraine cases and controls. Furthermore, both for preventive medicines (i.e., amitriptyline, flunarizine, propranolol, topiramate, and valproic acid) and relief treatment of migraine (i.e., ergotamine), case patients have significant higher proportions of utilization than control samples. However, for propranolol, topiramate, and valproic acid, case patients have lower exposure dosages and durations. It is observed that the mean prescription dosage of migraine medication in the current study follows the corresponding DDD (≤1 DDD per day).

**Table 1:** Demographics of the dataset

Variable	Migraine (*n* = 19,356) (%)	Control (*n* = 96,780) (%)	*p* value
Male	5,328 (27.5)	26,640 (27.5)	1.000
Follow-up migraine	3,664 (18.9)	0	<0.001
Age (years)			
≤50	13,530 (69.9)	67,650 (69.9)	1.000
51–64	3,724 (19.2)	18,620 (19.2)	
≥65	2,102 (10.9)	10,510 (10.9)	
Drug medication			
Amitriptyline	211 (1.1)	335 (0.3)	<0.001
Dosage (mg) (SD)	1,506.3 (3,101)	2,393.1 (6,903.6)	0.073
Duration (day) (SD)	54.6 (90.4)	73.6 (153.1)	0.096
Dosage (DDD) (SD)	20.4 (41.8)	33.5 (96.0)	0.056
Average dosage (DDD) (SD)	0.4 (0.2)	0.5 (0.5)	0.01
Flunarizine	2,533 (13.1)	2,406 (2.5)	<0.001
Dosage (mg) (SD)	375.2 (704.0)	301.7 (796.8)	<0.001
Duration (day) (SD)	49.3 (92.4)	34.8 (86.9)	<0.001
Dosage (DDD) (SD)	37.5 (70.4)	30.2 (79.7)	<0.001
Average dosage (DDD) (SD)	0.9 (0.5)	1.1 (0.6)	<0.001
Propranolol	6,626 (34.2)	10,011 (10.3)	<0.001
Dosage (mg) (SD)	2,359.9 (5,604.7)	2,746.3 (6,607.6)	<0.001
Duration (day) (SD)	89.4 (175.6)	109.4 (216.2)	<0.001
Dosage (DDD) (SD)	14.8 (35.5)	17.2 (41.4)	<0.001
Average dosage (DDD) (SD)	0.2 (0.1)	0.2 (0.2)	0.003
Topiramate	428 (2.2)	96 (0.1)	<0.001
Dosage (mg) (SD)	9,419.6 (45,071.2)	42,088.8 (79,385.5)	<0.001
Duration (day) (SD)	87.6 (156.0)	283.2 (405.7)	<0.001
Dosage (DDD) (SD)	31.4 (150.3)	140.3 (264.6)	<0.001
Average dosage (DDD) (SD)	0.2 (0.2)	0.4 (0.4)	<0.001
Valproic acid	113 (0.6)	136 (0.1)	<0.001
Dosage (mg) (SD)	54,053.5 (116,266.7)	93,832.7 (132,227.4)	0.013
Duration (day) (SD)	91.0 (150.3)	140.3 (201.6)	0.033
Dosage (DDD) (SD)	36.0 (77.5)	62.6 (88.2)	0.013
Average dosage (DDD) (SD)	0.4 (0.3)	0.5 (0.3)	0.002
Ergotamine	6,088 (31.5)	2,575 (2.7)	<0.001
Dosage (mg) (SD)	57.4 (184.1)	21.5 (81.9)	<0.001
Duration (day) (SD)	37.0 (94.2)	13.5 (46.5)	<0.001
Dosage (DDD) (SD)	14.8 (46.7)	6.7 (27.8)	<0.001
Average dosage (DDD) (SD)	0.5 (0.3)	0.6 (0.6)	<0.001

**Fig. 1:**
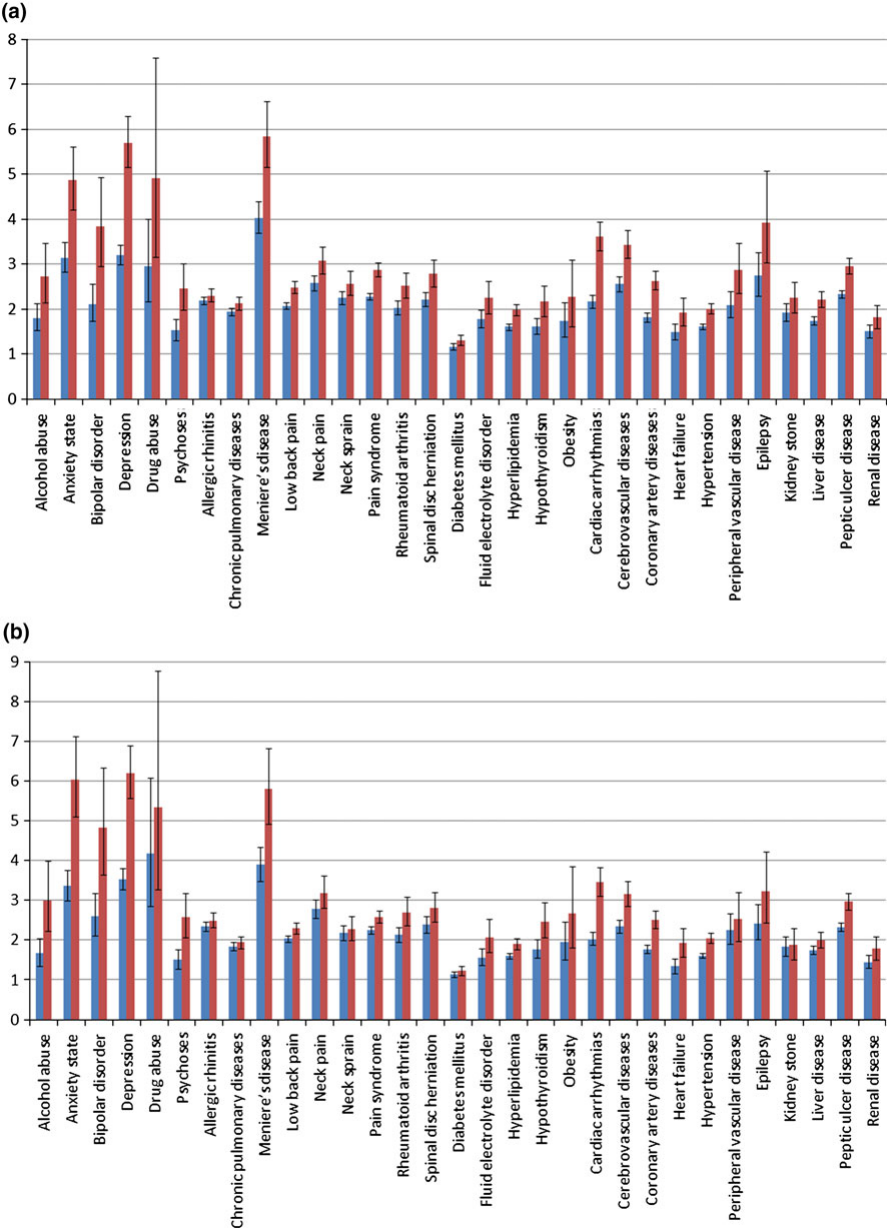
Relative risks of co-morbidities among migraine cases and controls **a** for the study period of 24 months before the index date, and **b** for the study period of 12 months after the index date. The *blue bars* represent values for original subjects, and the *red bars* represent values for samples of interest (color figure online)

Figure 1 shows the results obtained with the conventional analysis procedure, i.e., without invoking the proposed density estimation-based procedure. The blue bars show the relative risks of suffering co-morbidities among migraine cases and controls. The odds ratios with respect to the following co-morbidities are: alcohol abuse 1.8/1.67, anxiety state 3.14/3.36, bipolar disorder 2.11/2.6, depression 3.2/3.53, drug abuse 2.96/4.17, psychoses 1.53/1.5, allergic rhinitis 2.19/2.34, chronic pulmonary disease 1.94/1.84, Meniere’s disease 4.03/3.89, low back pain 2.07/2.04, neck pain 2.58/2.78, neck sprain 2.25/2.18, pain syndrome 2.28/2.25, rheumatoid arthritis 2.03/2.13, spinal disk herniation 2.21/2.39, diabetes mellitus 1.16/1.15, fluid electrolyte disorder 1.78/1.56, hyperlipidemia 1.6/1.6, hypothyroidism 1.61/1.77, obesity 1.73/1.94, cardiac arrhythmias 2.17/2.03, cerebrovascular disease 2.55/2.34, coronary artery disease 1.82/1.77, heart failure 1.49/1.34, hypertension 1.6/1.61, peripheral vascular disease 2.09/2.25, epilepsy 2.74/2.42, kidney stone 1.92/1.83, liver disease 1.74/1.74, peptic ulcer disease 2.33/2.33, and renal disease 1.5/1.45. The data presented in Fig. 1 reveal that migraine patients were more likely than age- and sex-matched controls to suffer these illnesses. Please refer to Supplementary Table 1 for more detailed statistics.

The red bars in Fig. 1 with the detailed data in Supplementary Table 2 show the relative risks of suffering co-morbidities between the migraine cases classified as samples of interest during the first stage of the proposed analysis procedure and their age- and sex-matched controls. In this respect, the RVKDE algorithm identified 7,146 migraine patients as samples of interest. Based on the data shown in Fig. 1 and the statistics shown in Supplementary Tables 1 and 2, we can conclude that those migraine cases of interest suffered even higher risks of co-morbidities.

**Fig. 2:**
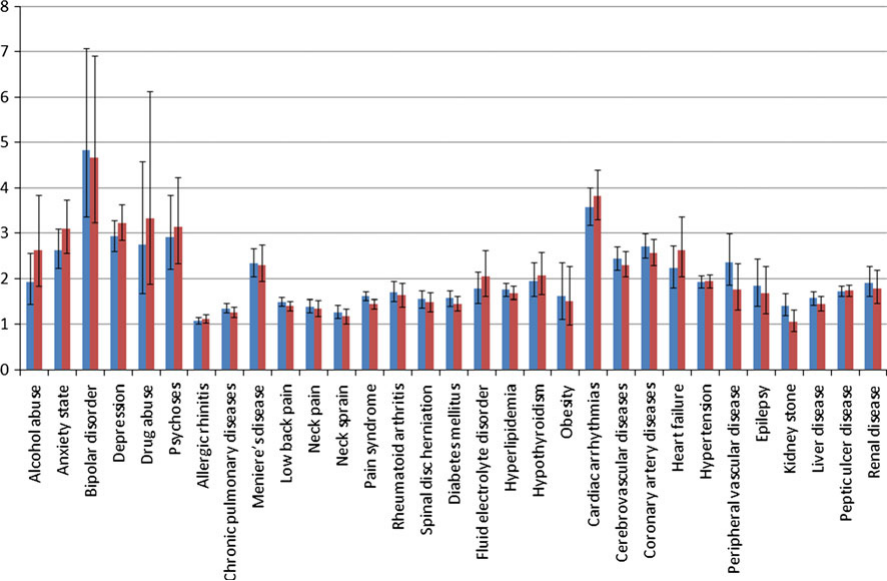
Relative risks of co-morbidities among cases of interest and the remaining migraine cases for the study period of 24 months before the index date (*blue bars*), and for the study period of 12 months after the index date (*red bars*) (color figure online)

According to the demographics shown in Table 2, the 7,146 cases of interest have lower male proportion than the remaining 12,210 migraine cases (24.8 vs. 29.1 %; *p* < 0.001). Moreover, the mean age of the cases of interest is older than the mean age of the remaining migraine cases, 45.3 versus 41.8 with *p* value <0.001. For both preventive medicines and relief treatment of migraine, cases of interest have significant higher utilization proportions than the remaining migraine patients. However, for topiramate and valproic acid, the cases of interest have lower exposure dosages and durations. Figure 2 and Supplementary Table 3 show the relative risks of co-morbidities among the cases of interest and the remaining migraine cases. We observed that the cases of interest suffered higher risks of co-morbidities than the remaining migraine patients.

**Table 2:** Demographics among cases of interest and the remaining migraine cases

Variable	Of interest (*n* = 7,146) (%)	Remaining (*n* = 12,210) (%)	*p* value
Male	1,773 (24.8)	3,555 (29.1)	<0.001
Follow-up migraine	1,636 (22.9)	2,028 (16.6)	<0.001
Age (years)			
≤50	4,598 (64.3)	8,932 (73.2)	<0.001
51–64	1,572 (22.0)	2,152 (17.6)	
≥65	976 (13.7)	1,126 (9.2)	
Drug medication			
Amitriptyline	205 (2.9)	16 (0.1)	<0.001
Dosage (mg) (SD)	1,444.0 (2,534.8)	2,304.7 (7,274.7)	0.286
Duration (day) (SD)	54.7 (89.0)	53 (109.3)	0.943
Dosage (DDD) (SD)	19.6 (34.4)	30.7 (97.0)	0.304
Average dosage (DDD) (SD)	0.4 (0.2)	0.3 (0.2)	0.239
Flunarizine	2,362 (33.1)	171 (1.4)	<0.001
Dosage (mg) (SD)	396.8 (722.5)	76.6 (189.9)	<0.001
Duration (day) (SD)	52.0 (94.8)	12.1 (29.5)	<0.001
Dosage (DDD) (SD)	39.7 (72.3)	7.7 (19.0)	<0.001
Average dosage (DDD) (SD)	0.9 (0.5)	0.9 (0.5)	0.411
Propranolol	5,840 (81.7)	786 (6.4)	<0.001
Dosage (mg) (SD)	2,540.6 (5,866.7)	1,017.7 (2,654.5)	<0.001
Duration (day) (SD)	92.8 (177.1)	63.4 (162.2)	<0.001
Dosage (DDD) (SD)	15.9 (37.2)	6.4 (16.6)	<0.001
Average dosage (DDD) (SD)	0.181 (0.129)	0.152 (0.098)	<0.001
Topiramate	421 (5.9)	7 (0.1)	<0.001
Dosage (mg) (SD)	8,822.9 (42,984.2)	45,307.1 (117,072.4)	0.034
Duration (day) (SD)	86.9 (153.2)	127.8 (295.7)	0.492
Dosage (DDD) (SD)	29.4 (143.3)	151.0 (390.2)	0.034
Average dosage (DDD) (SD)	0.2 (0.2)	0.5 (0.5)	<0.001
Valproic acid	104 (1.5)	9 (0.1)	<0.001
Dosage (mg) (SD)	43,163.0 (86,795.3)	179,900 (269,999.2)	<0.001
Duration (day) (SD)	78.7 (117.7)	232.8 (336.8)	0.003
Dosage (DDD) (SD)	28.8 (57.9)	119.9 (180)	<0.001
Average dosage (DDD) (SD)	0.3 (0.3)	0.6 (0.2)	0.001
Ergotamine	2,902 (40.6)	3,186 (26.1)	<0.001
Dosage (mg) (SD)	71.4 (204.1)	44.6 (162.7)	<0.001
Duration (day) (SD)	47.6 (105.4)	27.3 (81.5)	<0.001
Dosage (DDD) (SD)	18.3 (51.5)	11.5 (41.5)	<0.001
Average dosage (DDD) (SD)	0.4 (0.3)	0.5 (0.3)	<0.001

Since Figs. 1 and 2 (and Supplementary Tables 2, 3) confirm that the first stage of the proposed analysis procedure successfully identified a subset of migraine cases who suffered higher risks of developing co-morbidities according to characteristics of medication exposure, it is highly desirable to conduct an in-depth analysis. Accordingly, in the second stage of the proposed analysis procedure, the G^2^DE algorithm was invoked to identify the main clusters among the 7,146 cases of interest. As mentioned earlier, before invoking the G^2^DE algorithm, factor analysis was carried out to identify the most informative features. In this respect, it must be noted that the set of cases of interest passed the two criteria commonly adopted to measure the adequacy of applying factor analysis. In fact, applying the Kaiser–Meyer–Olkin (KMO) test on the set of cases of interest yielded a value of 0.502, which is higher than the commonly adopted threshold of 0.5, and applying the Bartlett’s test yielded a value smaller than 0.001, which is significant for variance homogeneity. The end result of the factor analysis is that exposure dosages (in unit of milligram) for the five preventive medicines of migraine: amitriptyline, flunarizine, propranolol, topiramate, and valproic acid, were selected respectively.

**Fig. 3:**
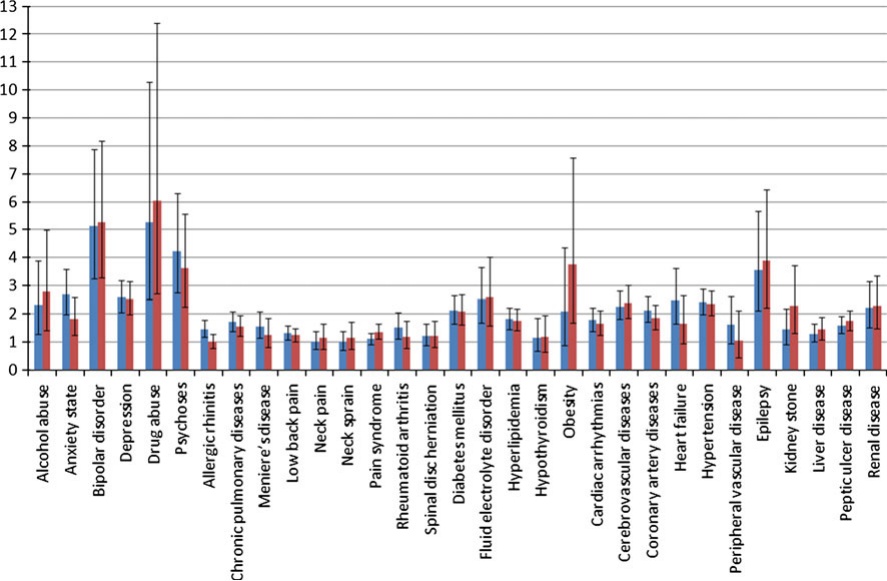
Relative risks of co-morbidities among the clusters identified by G^2^DE for the study period of 24 months before the index date (*blue bars*), and for the study period of 12 months after the index date (*red bars*) (color figure online)

The G^2^DE algorithm identified two clusters with distinctive characteristics shown in Table 3. Comparing the cases in cluster 0 and cluster 1, we can find that the cases in cluster 1 were generally older (52.5 vs. 44.7 with *p* value <0.001) but they have almost the same gender distribution. Furthermore, for both preventive medicines and relief treatment of migraine attacks, the case samples in cluster 1 had significant larger exposure dosages and longer durations. According to the results shown in Fig. 3 and Supplementary Table 4, cases in cluster 1 had higher risks of suffering mental disorders [odds ratio (OR): alcohol abuse 2.31/2.77, anxiety state 2.68/1.81, bipolar disorder 5.12/5.27, depression 2.57/2.5, drug abuse 5.26/6.02, and psychoses 4.22/3.61], diabetes mellitus (OR = 2.1/2.09), fluid electrolyte disorder (OR = 2.51/2.59), and cardiovascular/neurological diseases (OR: cardiac arrhythmias 1.77/1.63, cerebrovascular disease 2.26/2.38, coronary artery disease 2.11/1.83, hypertension 2.4/2.34, and epilepsy 3.55/3.88).

**Table 3:** Demographics among the clusters identified by G^2^DE

Variable	Cluster 1 (*n* = 489) (%)	Cluster 0 (*n* = 6,657) (%)	*p* value
Male	134 (27.4)	1,639 (24.6)	0.169
Follow-up migraine	153 (31.3)	1,483 (22.3)	<0.001
Age (years)			
≤50	216 (44.2)	4,382 (65.8)	<0.001
51–64	167 (34.2)	1,405 (21.1)	
≥65	106 (21.7)	870 (13.1)	
Drug medication			
Amitriptyline	194 (39.7)	11 (0.2)	<0.001
Dosage (mg) (SD)	1,524.0 (2,582.9)	32.5 (17.0)	0.057
Duration (day) (SD)	57.6 (90.7)	3 (1.2)	0.048
Dosage (DDD) (SD)	20.6 (35.0)	0.4 (0.2)	0.058
Average dosage (DDD) (SD)	0.4 (0.2)	0.1 (0.1)	0.001
Flunarizine	171 (35.0)	2,191 (32.9)	0.351
Dosage (mg) (SD)	2,055.4 (1,692.5)	267.4 (330.0)	<0.001
Duration (day) (SD)	255.6 (220.8)	36.1 (49.2)	<0.001
Dosage (DDD) (SD)	205.5 (169.2)	26.7 (33.0)	<0.001
Average dosage (DDD) (SD)	0.9 (0.4)	0.9 (0.5)	0.499
Propranolol	373 (76.3)	5,467 (82.1)	0.001
Dosage (mg) (SD)	15,310.1 (16,078.8)	1,669.3 (2,699.2)	<0.001
Duration (day) (SD)	438.2 (399.6)	69.3 (118.0)	<0.001
Dosage (DDD) (SD)	96.6 (102.8)	10.4 (16.9)	<0.001
Average dosage (DDD) (SD)	0.233 (0.175)	0.177 (0.124)	<0.001
Topiramate	50 (10.2)	371 (5.6)	<0.001
Dosage (mg) (SD)	44,320.5 (117,333.2)	4,038.8 (8,993.7)	<0.001
Duration (day) (SD)	236.3 (301.4)	66.8 (105.8)	<0.001
Dosage (DDD) (SD)	147.7 (391.1)	13.5 (30.0)	<0.001
Average dosage (DDD) (SD)	0.327 (0.344)	0.181 (0.192)	<0.001
Valproic acid	33 (6.7)	71 (1.1)	<0.001
Dosage (mg) (SD)	112,018.2 (129,297.2)	11,159.9 (12,984.2)	<0.001
Duration (day) (SD)	181.3 (160.8)	31.0 (36.2)	<0.001
Dosage (DDD) (SD)	74.7 (86.2)	7.4 (8.7)	<0.001
Average dosage (DDD) (SD)	0.5 (0.3)	0.3 (0.2)	0.002
Ergotamine	203 (41.5)	2,699 (40.5)	0.674
Dosage (mg) (SD)	170.3 (453.5)	63.9 (169.2)	<0.001
Duration (day) (SD)	101.3 (174.8)	43.6 (97.1)	<0.001
Dosage (DDD) (SD)	43.6 (113.6)	16.4 (42.8)	<0.001
Average dosage (DDD) (SD)	0.5 (0.4)	0.4 (0.3)	0.24

## Discussions

### Co-morbidities of migraine

According to the results shown in Fig. 1 and Supplementary Table 1, our study confirms co-morbid relationships between migraine and various diseases even without carrying out the screening process to identify samples of interest. In our study, the diseases included for co-morbidity analysis can be classified into six categories.

#### Mental disorders

The correlation between mental disorder and migraine has been studied extensively in recent years and our results match the previous observations. The American Migraine Prevalence and Prevention (AMPP) study demonstrated that both depression (OR = 2.0) and anxiety (OR = 1.8) were included in the co-morbidity profiles of chronic migraine and episodic migraine patients (Buse et al. 2010). Based on the Italian version of the Mini International Neuropsychiatry Interview (MINI), Beghi et al. (2010) reported that significant proportions of depression and moderate proportions of anxiety were among migraine and tension-type headache patients. Dilsaver et al. (2009) showed the association between bipolar disorder and migraine by observing that patients with a family history of bipolar disorder were 4.38 (OR = 4.38) times more likely to have migraine headaches than those without. A recent questionnaire survey revealed that migraine was far more prevalent in the substance abusers, e.g., alcohol, benzodiazepine, or opioids (Beckmann et al. 2012). Because of distinctness for study designs and data sources, we might not directly compare our quantitative results with benchmark values from literatures. Nevertheless, our results in Fig. 1 and Supplementary Table 1 confirm that migraine patients are more likely than controls to suffer mental disorders, which is in conformity with the observations reported in previous studies. Shared serotonergic dysfunction between migraine and affective disorders may contribute these associations.

#### Otolaryngology

The association between migraine and asthma has still been under debate. The Head-HUNT study showed that both migraine and non-migrainous headache were 1.5 times (OR = 1.5) more prevalent among those with asthma than those without (Aamodt et al. 2007). On the contrary, another study showed that the risk of developing follow-up incident asthma was not materially higher for migraine patients (Becker et al. 2008). Our results support the co-morbid associations between migraine and allergic rhinitis (OR = 2.19/2.34) as well as chronic pulmonary disease (OR = 1.94/1.84). Recent evidence has suggested that activation and sensitization of primary afferent meningeal nociceptive neurons trigger migraine attacks and the triggering factor is the involvement of mast cells (Levy et al. 2006). These findings may explain why allergic nasal symptoms accompany migraine. Finally, it has been reported that patients with Meniere’s disease suffered higher prevalence of migraine and Meniere’s disease patients with migraine suffered more severe vertigo or hearing loss (Cha et al. 2007). Again, the results from our population-based study are in conformity with these findings.

#### Musculoskeletal illnesses

The Nord-Trondelag Health Survey found that prevalence of chronic headache was 4.6 times (OR = 4.6) higher among individuals with musculoskeletal symptoms than among those without (Hagen et al. 2002). Similarly, 92 Israeli consecutive patients with migraine from a tertiary headache clinic suffered high incidence of fibromyalgia syndrome (Ifergane et al. 2006). In addition, the National Health Examination and Nutrition Survey (NHANES) showed adults with headache/migraine suffered increased odds of rheumatoid arthritis (OR = 1.95) (Kalaydjian and Merikangas 2008). Our results in Fig. 1 and Supplementary Table 1 confirm the co-morbid associations between migraine and various musculoskeletal illnesses.

#### Metabolism and endocrinology

Results of any significant association between migraine and diabetes are conflicting: some showed co-morbidity (OR = 1.4) (Bigal et al. 2010), some not (Le et al. 2011), and yet the other reported an inverse association (Burn et al. 1984). This debate may be why our results only show a slight co-morbid association between migraine and diabetes mellitus (OR = 1.16/1.15). Similarly, the International Headache Society (IHS) Classification of Headache Disorders Second Edition includes “Headache attributed to hypothyroidism”, and it was observed that approximately 30 % of 102 hypothyroid patients had bilateral, continuous headache (Moreau et al. 1998). Our observations also support this conclusion (OR = 1.61/1.77), but another population-based study obtained a conflicting result with negative correlation (OR = 0.5) (Hagen et al. 2001). Elevated levels of cholesterol (OR = 5.97) and triglycerides (OR = 4.42) had ever been reported to be associated with migraine (Rist et al. 2011), but there is no direct significant association between electrolyte imbalance and migraine as far as we are concerned to support our results (OR = 1.78/1.56). Finally, one epidemiologic study found the positive association between migraine and obesity (Peterlin et al. 2010). This suggestion is also supported by our analyses (OR = 1.73/1.94) while another population-based study disputed the association (OR = 1.03) (Winter et al. 2009).

#### Cardiovascular and neurological diseases

For over one decade, it has been a consensus among biomedical scientists that migraine increases atherosclerosis risk and ignites cardiovascular disorders such as instance angina, ischemic heart disease (OR = 1.94–2.2), and stroke (OR = 1.5–5.46) (Bigal et al. 2010; Kurth et al. 2008; Stang et al. 2005). Schurks et al. (2008) suggested that the MTHFR 677TT genotype magnifies risk of cardiovascular disease among migraine patients. Bigal et al. (2010) demonstrated a higher cardiovascular risk profile among migraine patients with higher cholesterol and blood pressure level. On the other hand, the co-morbidity between migraine and epilepsy has been suggested in one recent Dutch study (OR = 1.39) (Nuyen et al. 2006). The linkage between epilepsy and visual aura migraine possibly results from a gene defect located at chromosome 9q21–q22 (Deprez et al. 2007). In our population-based study, all these cardiovascular/neurological illnesses were prevalent among migraine patients than among matched controls.

#### Gastroenterology and hepatology

One recent study has concluded that kidney stone is a co-morbidity of migraine (OR = 1.43) (Le et al. 2011), which coincides with our analyses (OR = 1.92/1.83). It was suggested that topiramate dosage, which is commonly used for migraine preventive treatment, was inversely correlated to urinary citrate excretion and led to increased risk of stone-forming (Kaplon et al. 2011). On the other hand, *Helicobacter* *pylori* infection might be both causes of hepatic encephalopathy and migraine symptoms in patients with cirrhosis (Hong et al. 2007). Although non-steroidal anti-inflammatory drugs, which are the symptomatic relief of headache and migraine, may be ulcer-causing medications, peptic ulcer disease did not have a high prevalence in the US headache patients (Rozen and Fishman 2012). This is contradictory to our observations for the co-morbid relation between migraine and peptide-ulcer disease (OR = 2.33), and prescriptions for drugs of headache relief without the side effect of ulcer may explain this difference. Finally, increased plasma concentrations of endothelin-1 had been described in both migraine and renal disease patients; this might be the reason for their co-morbid association (Noll et al. 1996).

### Analysis results of density estimation

The co-morbid associations of migraine and various kinds of illnesses can be observed in Fig. 1 and Supplementary Table 1. However, no matter comparing the 7,146 migraine patients of interest extracted by RVKDE to their 35,730 age- and sex-matched controls, or to the remaining 12,210 migraine cases, they were even more likely to suffer these co-morbid illnesses. Our study verifies the effectiveness of density estimation algorithms on medical information analyses. The extracted migraine “patients of interest” had higher utilization proportions of both preventive medicines and relief treatment for migraine than the filtered cases. Because migraine is a common chronic, recurrent condition, it is believed that patients with significant medication utilization are more representative for this disease. Since some of the co-morbid illnesses studied belong to the Charlson (Charlson et al. 1987) or Elixhauser index (Elixhauser et al. 1998), it is suggested that physicians screen these patients for further risks of poor health conditions.

Moreover 489 of the 7,146 migraine cases of interest could be identified by G^2^DE according to the characteristics of medication exposures for migraine. Although for flunarizine and ergotamine, the selected 489 cases and the remaining 6,657 ones did not show significant differences in utilization proportions, these migraine patients had larger exposure dosages and longer durations for all kinds of drugs studied. This can be treated as a migraine severity measurement. According to the results shown in Fig. 3 and Supplementary Table 4, exposure dosage/duration of medicines discriminates best for the mental disorders and cardiovascular/neurological diseases. It was observed that the worse the pain profile, the worse the physical functioning and mental health (Wang et al. 2001). So our results are in conformity with the previous conclusions.

Although conventional algorithms of regression analysis are applicable for data mining in medical and/or clinical information, they borrow the idea from multi-dimensional contingency table to determine certain associations between the dependent variable and the risk factors. Rather than fitting a more saturated model, it might be more inclined to reflect an interaction structure between the dependent variables and corresponding risk factors. However, in this research, we would like to refer the concept of discriminate analysis: classifying an object that comes from one of two populations having associated densities *f1* and *f2* could be based upon the likelihood ratio *f1*/*f2*. It is expected that the significant difference between density distributions represents variances of the dependent variables in distinct groups of independent variable, e.g., an overall migraine severity measurement quantified by synergistic medication exposures. In fact, we ever categorized the migraine patients of interest as the contingency table by age, but this clustering cannot discriminate mental disorders the way G^2^DE can (data are not shown). So the proposed density estimation-based analysis procedure conceivably provides valuable insights which might be overlooked by conventional methods.

### Limitations

A major strength of our study was utilization of a large population-based medical claims database, but there were some limitations. First, administrative claims reported by hospitals or clinics may be less accurate than clinical diagnoses and observer-rating scales. Second, prescriptions of medications for migraine do not guarantee drug adherence. Third, the administrative claims data of NHIRD did not include detailed personal information like body mass index, living habits, or results of laboratory tests, which might be important confounding factors. Finally, more confounding factors of the outcome diseases, e.g., age, sex, medication drugs, treatment procedures, or associated symptoms, should be taken into account.

## Conclusions

In recent years, data analysis based on large medical and clinical databases has gained attention among biomedical researchers. Furthermore, scientists have turned to exploit advanced machine learning and/or data mining approaches to extract valuable clues hidden in large medical and clinical databases. In this paper, we have proposed a density estimation-based data analysis procedure to investigate the co-morbid associations between migraine and the suspected diseases by characteristics of medication exposure. The primary objective of this study is to develop a novel analysis procedure that can discover insightful knowledge from large medical databases. The results obtained by applying the proposed two-staged procedure to analyze co-morbidities of migraine reveal that the proposed procedure can effectively identify a number of clusters of cases with distinctive characteristics. Furthermore, it has been observed that the distinctive characteristics of the clusters are in conformity with the recently discovered knowledge in biomedical research. Accordingly, it is conceivable that the proposed analysis procedure will be exploited to provide valuable clues of pathogenesis and facilitate development of proper treatment strategies.

Three further courses are undertaken. Firstly, since effectiveness of the proposed analysis procedure has been verified, this method will be exploited to investigate characteristics of more epidemics, such as osteoporosis or herpes zoster. Secondly, appropriate statistical tests will be issued on the mined facts to strengthen persuasiveness of this approach. Finally, application of various advanced machine learning/data mining algorithms on medical and/or clinical databases will also be studied.

## Electronic supplementary material

Below is the link to the electronic supplementary material.

Supplementary material 1 (PDF 238 kb)
