# Supplementary material for: Application of density estimation algorithms in analyzing co-morbidities of migraine
Source: Netw Model Anal Health Inform Bioinform. 2013 Feb 12;2(2):95–107. doi: 10.1007/s13721-013-0028-8 (PMC3873085; doi:10.1007/s13721-013-0028-8)
Supplement: Supplementary file 1 — Supplementary material 1 (PDF 238 kb) [file 13721_2013_28_MOESM1_ESM.pdf]

Application of Density Estimation Algorithms in Analyzing Co-morbidities of Migraine

Meng-Han Yang, PhD[1]; Fu-Yi Yang, MD[2]; Yen-Jen Oyang, PhD[3]

1. *Department of Computer Science and Information Engineering, National Kaohsiung University of Applied Sciences, No. 415 Chien Kung Rd., Kaohsiung City 80778, Taiwan, ROC*
2. *The Department of Neurology, Taipei Tzu Chi General Hospital, No.289, Jianguo Rd., Xindian Dist., New Taipei City 23142, Taiwan, ROC*
3. *Department of Computer Science and Information Engineering, National Taiwan University, No. 1, Sec. 4, Roosevelt Rd., Taipei City 10617, Taiwan, ROC*

menghanyang@kuas.edu.tw; fuyi127@yahoo.com.tw; yjoyang@csie.ntu.edu.tw

The manuscript is submitted to Network Modeling Analysis in Health Informatics and Bioinformatics with article ID NHIB-D-12-00025.

Supplementary table 1. Relative risks of co-morbidities among migraine cases and controls (a) for the study period of 24 months before the index date, and (b) for the study period of 12 months after the index date

(a)

Variable	Migraine (n=19,356) (%)	Control* (n=96,780) (%)	OR (95% CI)	P value
Alcohol abuse	199 (1.0)	555 (0.6)	1.8 (1.53-2.12)	<0.001
Anxiety state	606 (3.1)	986 (1.0)	3.14 (2.83-3.48)	<0.001
Bipolar disorder	144 (0.7)	343 (0.4)	2.11 (1.73-2.56)	<0.001
Depression	1,376 (7.1)	2,261 (2.3)	3.2 (2.99-3.43)	<0.001
Drug abuse	65 (0.3)	110 (0.1)	2.96 (2.17-4.01)	<0.001
Psychoses	217 (1.1)	712 (0.7)	1.53 (1.31-1.78)	<0.001
Allergic rhinitis	4,069 (21)	10,486 (10.8)	2.19 (2.1-2.28)	<0.001
Chronic pulmonary diseases	3,385 (17.5)	9,543 (9.9)	1.94 (1.86-2.02)	<0.001
Meniere’s disease	942 (4.9)	1,213 (1.3)	4.03 (3.7-4.39)	<0.001
Low back pain	6,939 (35.8)	20,575 (21.3)	2.07 (2-2.14)	<0.001
Neck pain	1,497 (7.7)	3,040 (3.1)	2.58 (2.42-2.75)	<0.001
Neck sprain	1,379 (7.1)	3,188 (3.3)	2.25 (2.11-2.4)	<0.001
Pain syndrome	7,148 (36.9)	19,760 (20.4)	2.28 (2.21-2.36)	<0.001
Rheumatoid arthritis	1,026 (5.3)	2,602 (2.7)	2.03 (1.88-2.18)	<0.001
Spinal disc herniation	1,176 (6.1)	2,748 (2.8)	2.21 (2.06-2.37)	<0.001
Diabetes mellitus	1,592 (8.2)	6,926 (7.2)	1.16 (1.1-1.23)	<0.001
Fluid electrolyte disorder	421 (2.2)	1,197 (1.2)	1.78 (1.59-1.98)	<0.001
Hyperlipidemia	2,914 (15.1)	9,650 (10.0)	1.6 (1.53-1.67)	<0.001
Hypothyroidism	439 (2.3)	1,378 (1.4)	1.61 (1.44-1.79)	<0.001
Obesity	109 (0.6)	316 (0.3)	1.73 (1.38-2.14)	<0.001
Cardiac arrhythmias	1,366 (7.1)	3,275 (3.4)	2.17 (2.03-2.31)	<0.001
Cerebrovascular diseases	1,539 (8.0)	3,170 (3.3)	2.55 (2.39-2.72)	<0.001
Coronary artery diseases	1,825 (9.4)	5,249 (5.4)	1.82 (1.72-1.92)	<0.001
Heart failure	371 (1.9)	1,251 (1.3)	1.49 (1.33-1.68)	<0.001

Hypertension	3,933 (20.3)	13,276 (13.7)	1.6 (1.54-1.67)	<0.001
Peripheral vascular disease	281 (1.5)	677 (0.7)	2.09 (1.82-2.4)	<0.001
Epilepsy	201 (1.0)	369 (0.4)	2.74 (2.3-3.25)	<0.001
Kidney stone	540 (2.8)	1,423 (1.5)	1.92 (1.74-2.12)	<0.001
Liver disease	2,222 (11.5)	6,715 (6.9)	1.74 (1.65-1.83)	<0.001
Peptic ulcer disease	4,627 (23.9)	11,502 (11.9)	2.33 (2.24-2.42)	<0.001
Renal disease	509 (2.6)	1,711 (1.8)	1.5 (1.36-1.66)	<0.001

OR, odds ratio; CI, confidence interval; *, as the reference

(b)

Variable	Migraine (n=19,356) (%)	Control* (n=96,780) (%)	OR (95% CI)	P value
Alcohol abuse	119 (0.6)	358 (0.4)	1.67 (1.35-2.04)	<0.001
Anxiety state	482 (2.5)	731 (0.8)	3.36 (2.99-3.77)	<0.001
Bipolar disorder	137 (0.7)	265 (0.3)	2.6 (2.11-3.19)	<0.001
Depression	1,170 (6.0)	1,731 (1.8)	3.53 (3.27-3.81)	<0.001
Drug abuse	50 (0.3)	60 (0.1)	4.17 (2.86-6.07)	<0.001
Psychoses	191 (1.0)	637 (0.7)	1.5 (1.28-1.77)	<0.001
Allergic rhinitis	2,982 (15.4)	6,999 (7.2)	2.34 (2.23-2.45)	<0.001
Chronic pulmonary diseases	2,197 (11.4)	6,290 (6.5)	1.84 (1.75-1.94)	<0.001
Meniere’s disease	551 (2.8)	724 (0.7)	3.89 (3.47-4.35)	<0.001
Low back pain	4,570 (23.6)	12,759 (13.2)	2.04 (1.96-2.11)	<0.001
Neck pain	872 (4.5)	1,616 (1.7)	2.78 (2.55-3.02)	<0.001
Neck sprain	801 (4.1)	1,883 (1.9)	2.18 (2-2.37)	<0.001
Pain syndrome	4,911 (25.4)	12,672 (13.1)	2.26 (2.17-2.34)	<0.001
Rheumatoid arthritis	679 (3.5)	1,625 (1.7)	2.13 (1.94-2.33)	<0.001
Spinal disc herniation	786 (4.1)	1,685 (1.7)	2.39 (2.19-2.6)	<0.001
Diabetes mellitus	1,399 (7.2)	6,165 (6.4)	1.15 (1.08-1.22)	<0.001
Fluid electrolyte disorder	260 (1.3)	836 (0.9)	1.56 (1.36-1.79)	<0.001
Hyperlipidemia	2,400 (12.4)	7,859 (8.1)	1.6 (1.53-1.68)	<0.001
Hypothyroidism	327 (1.7)	928 (1.0)	1.77 (1.56-2.01)	<0.001
Obesity	90 (0.5)	232 (0.2)	1.94 (1.52-2.47)	<0.001

Cardiac arrhythmias	928 (4.8)	2,338 (2.4)	2.03 (1.88-2.2)	<0.001
Cerebrovascular diseases	1,203 (6.2)	2,665 (2.8)	2.34 (2.18-2.51)	<0.001
Coronary artery diseases	1,410 (7.3)	4,106 (4.2)	1.77 (1.67-1.89)	<0.001
Heart failure	266 (1.4)	994 (1.0)	1.34 (1.17-1.54)	<0.001
Hypertension	3,570 (18.4)	11,896 (12.3)	1.61 (1.55-1.68)	<0.001
Peripheral vascular disease	194 (1.0)	433 (0.4)	2.25 (1.9-2.67)	<0.001
Epilepsy	168 (0.9)	349 (0.4)	2.42 (2.01-2.9)	<0.001
Kidney stone	316 (1.6)	870 (0.9)	1.83 (1.61-2.08)	<0.001
Liver disease	1,464 (7.6)	4,347 (4.5)	1.74 (1.64-1.85)	<0.001
Peptic ulcer disease	3,105 (16.0)	7,327 (7.6)	2.33 (2.23-2.44)	<0.001
Renal disease	396 (2.0)	1,371 (1.4)	1.45 (1.3-1.63)	<0.001

*, as the reference

Supplementary table 2. Relative risks of co-morbidities among migraine cases of interest and controls (a) for the study period of 24 months before the index date, and (b) for the study period of 12 months after the index date

(a)

**Variable**	**Migraine (n=7,146) (%)**	**Control* (n=35,730) (%)**	**OR (95% CI)**	**P value**
Alcohol abuse	105 (1.5)	194 (0.5)	2.73 (2.14-3.46)	<0.001
Anxiety state	362 (5.1)	388 (1.1)	4.86 (4.2-5.62)	<0.001
Bipolar disorder	106 (1.5)	140 (0.4)	3.83 (2.96-4.93)	<0.001
Depression	842 (11.8)	820 (2.3)	5.69 (5.15-6.28)	<0.001
Drug abuse	40 (0.6)	41 (0.1)	4.9 (3.16-7.59)	<0.001
Psychoses	136 (1.9)	280 (0.8)	2.46 (1.99-3.01)	<0.001
Allergic rhinitis	1,562 (21.9)	3,871 (10.8)	2.3 (2.16-2.46)	<0.001
Chronic pulmonary diseases	1,449 (20.3)	3,808 (10.7)	2.13 (1.99-2.28)	<0.001
Meniere’s disease	533 (7.5)	486 (1.4)	5.84 (5.15-6.63)	<0.001
Low back pain	2,982 (41.7)	7,999 (22.4)	2.48 (2.35-2.62)	<0.001
Neck pain	662 (9.3)	1,149 (3.2)	3.07 (2.78-3.39)	<0.001
Neck sprain	583 (8.2)	1,196 (3.3)	2.56 (2.31-2.84)	<0.001
Pain syndrome	3,148 (44.1)	7,695 (21.5)	2.87 (2.72-3.03)	<0.001

Rheumatoid arthritis	504 (7.1)	1,047 (2.9)	2.51 (2.25-2.8)	<0.001
Spinal disc herniation	549 (7.7)	1,040 (2.9)	2.78 (2.49-3.09)	<0.001
Diabetes mellitus	744 (10.4)	2,927 (8.2)	1.3 (1.2-1.42)	<0.001
Fluid electrolyte disorder	213 (3.0)	484 (1.4)	2.24 (1.9-2.63)	<0.001
Hyperlipidemia	1,409 (19.7)	3,951 (11.1)	1.98 (1.85-2.11)	<0.001
Hypothyroidism	232 (3.2)	547 (1.5)	2.16 (1.84-2.52)	<0.001
Obesity	53 (0.7)	118 (0.3)	2.26 (1.62-3.1)	<0.001
Cardiac arrhythmias	895 (12.5)	1,363 (3.8)	3.61 (3.3-3.94)	<0.001
Cerebrovascular diseases	876 (12.3)	1,399 (3.9)	3.43 (3.14-3.75)	<0.001
Coronary artery diseases	1,075 (15.0)	2,252 (6.3)	2.63 (2.44-2.84)	<0.001
Heart failure	208 (2.9)	551 (1.5)	1.91 (1.63-2.25)	<0.001
Hypertension	1,944 (27.2)	5,607 (15.7)	2.01 (1.89-2.13)	<0.001
Peripheral vascular disease	162 (2.3)	287 (0.8)	2.86 (2.35-3.47)	<0.001
Epilepsy	104 (1.5)	134 (0.4)	3.92 (3.03-5.07)	<0.001
Kidney stone	243 (3.4)	554 (1.6)	2.24 (1.92-2.6)	<0.001
Liver disease	1,032 (14.4)	2,531 (7.1)	2.21 (2.05-2.39)	<0.001
Peptic ulcer disease	2,163 (30.3)	4,571 (12.8)	2.96 (2.79-3.14)	<0.001
Renal disease	266 (3.7)	749 (2.1)	1.81 (1.56-2.08)	<0.001

*, as the reference

(b)

**Variable**	**Migraine (n=7,146) (%)**	**Control* (n=35,730) (%)**	**OR (95% CI)**	**P value**
Alcohol abuse	72 (1.0)	121 (0.3)	3 (2.23-4)	<0.001
Anxiety state	307 (4.3)	264 (0.7)	6.03 (5.11-7.13)	<0.001
Bipolar disorder	100 (1.4)	105 (0.3)	4.82 (3.65-6.34)	<0.001
Depression	744 (10.4)	658 (1.8)	6.19 (5.56-6.9)	<0.001
Drug abuse	33 (0.5)	31 (0.1)	5.34 (3.27-8.76)	<0.001
Psychoses	123 (1.7)	242 (0.7)	2.57 (2.06-3.19)	<0.001
Allergic rhinitis	1,169 (16.4)	2,598 (7.3)	2.49 (2.32-2.69)	<0.001
Chronic pulmonary	917 (12.8)	2,520 (7.1)	1.94 (1.79-2.1)	<0.001

diseases				
Meniere's disease	313 (4.4)	280 (0.8)	5.8 (4.93-6.83)	<0.001
Low back pain	1,969 (27.6)	5,079 (14.2)	2.3 (2.16-2.44)	<0.001
Neck pain	380 (5.3)	618 (1.7)	3.19 (2.8-3.63)	<0.001
Neck sprain	324 (4.5)	729 (2.0)	2.28 (1.99-2.6)	<0.001
Pain syndrome	2,131 (29.8)	5,066 (14.2)	2.57 (2.43-2.73)	<0.001
Rheumatoid arthritis	328 (4.6)	625 (1.7)	2.7 (2.36-3.09)	<0.001
Spinal disc herniation	361 (5.1)	663 (1.9)	2.81 (2.47-3.21)	<0.001
Diabetes mellitus	632 (8.8)	2,619 (7.3)	1.23 (1.12-1.34)	<0.001
Fluid electrolyte disorder	141 (2.0)	344 (1.0)	2.07 (1.69-2.52)	<0.001
Hyperlipidemia	1,148 (16.1)	3,268 (9.1)	1.9 (1.77-2.04)	<0.001
Hypothyroidism	178 (2.5)	366 (1.0)	2.47 (2.06-2.95)	<0.001
Obesity	42 (0.6)	79 (0.2)	2.67 (1.82-3.86)	<0.001
Cardiac arrhythmias	627 (8.8)	965 (2.7)	3.46 (3.12-3.84)	<0.001
Cerebrovascular diseases	674 (9.4)	1,145 (3.2)	3.15 (2.85-3.47)	<0.001
Coronary artery diseases	821 (11.5)	1,760 (4.9)	2.51 (2.3-2.73)	<0.001
Heart failure	160 (2.2)	421 (1.2)	1.92 (1.59-2.3)	<0.001
Hypertension	1,783 (25.0)	4,990 (14.0)	2.05 (1.93-2.18)	<0.001
Peripheral vascular disease	98 (1.4)	196 (0.5)	2.52 (1.97-3.21)	<0.001
Epilepsy	83 (1.2)	130 (0.4)	3.22 (2.43-4.23)	<0.001
Kidney stone	121 (1.7)	325 (0.9)	1.88 (1.52-2.31)	<0.001
Liver disease	662 (9.3)	1,733 (4.9)	2 (1.82-2.2)	<0.001
Peptic ulcer disease	1,489 (20.8)	2,908 (8.1)	2.97 (2.77-3.18)	<0.001
Renal disease	201 (2.8)	573 (1.6)	1.78 (1.51-2.09)	<0.001

*, as the reference

Supplementary table 3. Relative risks of co-morbidities among cases of interest and the remaining migraine cases (a) for the study period of 24 months before the index date, and (b) for the study period of 12 months after the index date

(a)

Variable	Of interest (n=7,146) (%)	Remaining* (n=12,210) (%)	OR (95% CI)	P value
Alcohol abuse	105 (1.5)	94 (0.8)	1.92 (1.45-2.55)	<0.001
Anxiety state	362 (5.1)	244 (2.0)	2.62 (2.22-3.09)	<0.001
Bipolar disorder	106 (1.5)	38 (0.3)	4.82 (3.36-7.08)	<0.001
Depression	842 (11.8)	534 (4.4)	2.92 (2.61-3.27)	<0.001
Drug abuse	40 (0.6)	25 (0.2)	2.74 (1.67-4.58)	<0.001
Psychoses	136 (1.9)	81 (0.7)	2.91 (2.21-3.84)	<0.001
Allergic rhinitis	1,562 (21.9)	2,507 (20.5)	1.08 (1.01-1.16)	0.029
Chronic pulmonary diseases	1,449 (20.3)	1,936 (15.9)	1.35 (1.25-1.46)	<0.001
Meniere's disease	533 (7.5)	409 (3.3)	2.33 (2.04-2.66)	<0.001
Low back pain	2,982 (41.7)	3,957 (32.4)	1.49 (1.41-1.59)	<0.001
Neck pain	662 (9.3)	835 (6.8)	1.39 (1.25-1.55)	<0.001
Neck sprain	583 (8.2)	796 (6.5)	1.27 (1.14-1.42)	<0.001
Pain syndrome	3,148 (44.1)	4,000 (32.8)	1.62 (1.52-1.72)	<0.001
Rheumatoid arthritis	504 (7.1)	522 (4.3)	1.7 (1.5-1.93)	<0.001
Spinal disc herniation	549 (7.7)	627 (5.1)	1.54 (1.37-1.73)	<0.001
Diabetes mellitus	744 (10.4)	848 (6.9)	1.56 (1.4-1.73)	<0.001
Fluid electrolyte disorder	213 (3.0)	208 (1.7)	1.77 (1.46-2.15)	<0.001
Hyperlipidemia	1,409 (19.7)	1,505 (12.3)	1.75 (1.61-1.89)	<0.001
Hypothyroidism	232 (3.2)	207 (1.7)	1.95 (1.61-2.35)	<0.001
Obesity	53 (0.7)	56 (0.5)	1.62 (1.11-2.36)	0.012
Cardiac arrhythmias	895 (12.5)	471 (3.9)	3.57 (3.18-4.01)	<0.001
Cerebrovascular diseases	876 (12.3)	663 (5.4)	2.43 (2.19-2.7)	<0.001
Coronary artery diseases	1,075 (15.0)	750 (6.1)	2.71 (2.45-2.99)	<0.001
Heart failure	208 (2.9)	163 (1.3)	2.22 (1.8-2.73)	<0.001
Hypertension	1,944 (27.2)	1,989 (16.3)	1.92 (1.79-2.06)	<0.001
Peripheral vascular disease	162 (2.3)	119 (1.0)	2.36 (1.86-3)	<0.001
Epilepsy	104 (1.5)	97 (0.8)	1.84 (1.4-2.44)	<0.001

Kidney stone	243 (3.4)	297 (2.4)	1.41 (1.19-1.68)	<0.001
Liver disease	1,032 (14.4)	1,190 (9.7)	1.56 (1.43-1.71)	<0.001
Peptic ulcer disease	2,163 (30.3)	2,464 (20.2)	1.72 (1.61-1.84)	<0.001
Renal disease	266 (3.7)	243 (2.0)	1.9 (1.6-2.27)	<0.001

*, as the reference

(b)

**Variable**	**Of interest (n=7,146) (%)**	**Remaining* (n=12,210) (%)**	**OR (95% CI)**	**P value**
Alcohol abuse	72 (1.0)	47 (0.4)	2.63 (1.83-3.83)	<0.001
Anxiety state	307 (4.3)	175 (1.4)	3.09 (2.56-3.73)	<0.001
Bipolar disorder	100 (1.4)	37 (0.3)	4.67 (3.23-6.9)	<0.001
Depression	744 (10.4)	426 (3.5)	3.21 (2.84-3.64)	<0.001
Drug abuse	33 (0.5)	17 (0.1)	3.33 (1.88-6.12)	<0.001
Psychoses	123 (1.7)	68 (0.6)	3.13 (2.33-4.23)	<0.001
Allergic rhinitis	1,169 (16.4)	1,813 (14.8)	1.12 (1.04-1.21)	0.005
Chronic pulmonary diseases	917 (12.8)	1,280 (10.5)	1.26 (1.15-1.38)	<0.001
Meniere's disease	313 (4.4)	238 (1.9)	2.3 (1.94-2.74)	<0.001
Low back pain	1,969 (27.6)	2,601 (21.3)	1.41 (1.31-1.5)	<0.001
Neck pain	380 (5.3)	492 (4.0)	1.34 (1.17-1.53)	<0.001
Neck sprain	324 (4.5)	477 (3.9)	1.17 (1.01-1.35)	0.035
Pain syndrome	2,131 (29.8)	2,780 (22.8)	1.44 (1.35-1.54)	<0.001
Rheumatoid arthritis	328 (4.6)	351 (2.9)	1.63 (1.39-1.89)	<0.001
Spinal disc herniation	361 (5.1)	425 (3.5)	1.48 (1.28-1.7)	<0.001
Diabetes mellitus	632 (8.8)	767 (6.3)	1.45 (1.3-1.61)	<0.001
Fluid electrolyte disorder	141 (2.0)	119 (1.0)	2.05 (1.6-2.62)	<0.001
Hyperlipidemia	1,148 (16.1)	1,252 (10.3)	1.68 (1.54-1.83)	<0.001
Hypothyroidism	178 (2.5)	149 (1.2)	2.07 (1.66-2.58)	<0.001
Obesity	42 (0.6)	48 (0.4)	1.5 (0.99-2.27)	0.056
Cardiac arrhythmias	627 (8.8)	301 (2.5)	3.81 (3.31-4.39)	<0.001
Cerebrovascular diseases	674 (9.4)	529 (4.3)	2.3 (2.04-2.59)	<0.001

Coronary artery diseases	821 (11.5)	589 (4.8)	2.56 (2.29-2.86)	<0.001
Heart failure	160 (2.2)	106 (0.9)	2.62 (2.05-3.36)	<0.001
Hypertension	1,783 (25.0)	1,787 (14.6)	1.94 (1.8-2.09)	<0.001
Peripheral vascular disease	98 (1.4)	96 (0.8)	1.75 (1.32-2.33)	<0.001
Epilepsy	83 (1.2)	85 (0.7)	1.68 (1.24-2.27)	<0.001
Kidney stone	121 (1.7)	195 (1.6)	1.06 (0.84-1.33)	0.610
Liver disease	662 (9.3)	802 (6.6)	1.45 (1.3-1.62)	<0.001
Peptic ulcer disease	1,489 (20.8)	1,616 (13.2)	1.73 (1.6-1.86)	<0.001
Renal disease	201 (2.8)	195 (1.6)	1.78 (1.46-2.18)	<0.001

*, as the reference

Supplementary table 4. Relative risks of co-morbidities among the clusters identified by G^2DE (a) for the study period of 24 months before the index date, and (b) for the study period of 12 months after the index date

(a)

**Variable**	**Cluster 1 (n=489) (%)**	**Cluster 0* (n=6,657) (%)**	**OR (95% CI)**	**P value**
Alcohol abuse	15 (3.1)	90 (1.4)	2.31 (1.28-3.9)	0.003
Anxiety state	56 (11.5)	306 (4.6)	2.68 (1.97-3.6)	<0.001
Bipolar disorder	28 (5.7)	78 (1.2)	5.12 (3.24-7.87)	<0.001
Depression	117 (23.9)	725 (10.9)	2.57 (2.05-3.2)	<0.001
Drug abuse	11 (2.2)	29 (0.4)	5.26 (2.5-10.3)	<0.001
Psychoses	31 (6.3)	105 (1.6)	4.22 (2.76-6.3)	<0.001
Allergic rhinitis	139 (28.4)	1,423 (21.4)	1.46 (1.19-1.79)	<0.001
Chronic pulmonary diseases	144 (29.4)	1,305 (19.6)	1.71 (1.39-2.09)	<0.001
Meniere’s disease	53 (10.8)	480 (7.2)	1.56 (1.15-2.09)	0.003
Low back pain	234 (47.9)	2,748 (41.3)	1.31 (1.09-1.57)	0.005
Neck pain	46 (9.4)	616 (9.3)	1.02 (0.73-1.38)	0.910
Neck sprain	40 (8.2)	543 (8.2)	1 (0.71-1.38)	0.986
Pain syndrome	226 (46.2)	2,922 (43.9)	1.1 (0.91-1.32)	0.318
Rheumatoid arthritis	49 (10.0)	455 (6.8)	1.52 (1.1-2.05)	0.008
Spinal disc herniation	44 (9.0)	505 (7.6)	1.2 (0.86-1.64)	0.258

Diabetes mellitus	91 (18.6)	653 (9.8)	2.1 (1.64-2.66)	<0.001
Fluid electrolyte disorder	32 (6.5)	181 (2.7)	2.51 (1.67-3.64)	<0.001
Hyperlipidemia	145 (29.7)	1,264 (19.0)	1.8 (1.46-2.2)	<0.001
Hypothyroidism	18 (3.7)	214 (3.2)	1.15 (0.68-1.83)	0.575
Obesity	7 (1.4)	46 (0.7)	2.09 (0.86-4.35)	0.072
Cardiac arrhythmias	95 (19.4)	800 (12.0)	1.77 (1.39-2.23)	<0.001
Cerebrovascular diseases	111 (22.7)	765 (11.5)	2.26 (1.8-2.82)	<0.001
Coronary artery diseases	127 (26.0)	948 (14.2)	2.11 (1.7-2.61)	<0.001
Heart failure	31 (6.3)	177 (2.7)	2.48 (1.64-3.62)	<0.001
Hypertension	223 (45.6)	1,721 (25.9)	2.4 (1.99-2.9)	<0.001
Peripheral vascular disease	17 (3.5)	145 (2.2)	1.62 (0.94-2.62)	0.065
Epilepsy	21 (4.3)	83 (1.2)	3.55 (2.13-5.68)	<0.001
Kidney stone	23 (4.7)	220 (3.3)	1.44 (0.91-2.19)	0.101
Liver disease	86 (17.6)	946 (14.2)	1.29 (1-1.63)	0.041
Peptic ulcer disease	196 (40.1)	1,967 (29.5)	1.59 (1.32-1.92)	<0.001
Renal disease	36 (7.4)	230 (3.5)	2.22 (1.52-3.15)	<0.001

*, as the reference

(b)

**Variable**	**Cluster 1 (n=489) (%)**	**Cluster 0* (n=6,657) (%)**	**OR (95% CI)**	**P value**
Alcohol abuse	12 (2.5)	60 (0.9)	2.77 (1.41-4.99)	0.001
Anxiety state	35 (7.2)	272 (4.1)	1.81 (1.24-2.57)	0.001
Bipolar disorder	27 (5.5)	73 (1.1)	5.27 (3.3-8.18)	<0.001
Depression	103 (21.1)	641 (9.6)	2.5 (1.98-3.15)	<0.001
Drug abuse	10 (2.0)	23 (0.3)	6.02 (2.72-12.4)	<0.001
Psychoses	25 (5.1)	98 (1.5)	3.61 (2.26-5.56)	<0.001
Allergic rhinitis	81 (16.6)	1,088 (16.3)	1.02 (0.79-1.29)	0.899
Chronic pulmonary diseases	88 (18.0)	829 (12.5)	1.54 (1.2-1.96)	<0.001
Meniere’s disease	26 (5.3)	287 (4.3)	1.25 (0.81-1.85)	0.295
Low back pain	154 (31.5)	1,815 (27.3)	1.23 (1-1.49)	0.044

Neck pain	29 (5.9)	351 (5.3)	1.13 (0.75-1.64)	0.532
Neck sprain	25 (5.1)	299 (4.5)	1.15 (0.74-1.71)	0.524
Pain syndrome	176 (36.0)	1,955 (29.4)	1.35 (1.11-1.64)	0.002
Rheumatoid arthritis	26 (5.3)	302 (4.5)	1.18 (0.77-1.75)	0.427
Spinal disc herniation	29 (5.9)	332 (5.0)	1.2 (0.8-1.75)	0.359
Diabetes mellitus	78 (16.0)	554 (8.3)	2.09 (1.61-2.69)	<0.001
Fluid electrolyte disorder	22 (4.5)	119 (1.8)	2.59 (1.59-4.03)	<0.001
Hyperlipidemia	119 (24.3)	1,029 (15.5)	1.76 (1.41-2.18)	<0.001
Hypothyroidism	14 (2.9)	164 (2.5)	1.17 (0.64-1.96)	0.585
Obesity	9 (1.8)	33 (0.5)	3.76 (1.68-7.58)	<0.001
Cardiac arrhythmias	64 (13.1)	563 (8.5)	1.63 (1.23-2.13)	<0.001
Cerebrovascular diseases	91 (18.6)	583 (8.8)	2.38 (1.86-3.02)	<0.001
Coronary artery diseases	90 (18.4)	731 (11.0)	1.83 (1.43-2.32)	<0.001
Heart failure	17 (3.5)	143 (2.1)	1.64 (0.95-2.66)	0.058
Hypertension	206 (42.1)	1,577 (23.7)	2.34 (1.94-2.83)	<0.001
Peripheral vascular disease	7 (1.4)	91 (1.4)	1.05 (0.44-2.11)	0.906
Epilepsy	18 (3.7)	65 (1.0)	3.88 (2.22-6.44)	<0.001
Kidney stone	17 (3.5)	104 (1.6)	2.27 (1.3-3.72)	0.002
Liver disease	61 (12.5)	601 (9.0)	1.44 (1.07-1.89)	0.012
Peptic ulcer disease	149 (30.5)	1,340 (20.1)	1.74 (1.42-2.12)	<0.001
Renal disease	28 (5.7)	173 (2.6)	2.28 (1.48-3.37)	<0.001

*, as the reference
